# Dalpiciclib induced hepatitis B virus reactivation leading to liver failure: a rare case report and review of the literature

**DOI:** 10.3389/fmed.2026.1769544

**Published:** 2026-03-18

**Authors:** Yuchen Peng, Yalin Xi, Aili Ding, Fanli Kong

**Affiliations:** Department of Pharmacy, Central Hospital of Dalian University of Technology, Dalian, China

**Keywords:** breast cancer, CDK4/6 inhibitors, clinical pharmacist, dalpiciclib, HBV reactivation, liver failure

## Abstract

Dalpiciclib is a cyclin-dependent kinase 4/6 (CDK4/6) inhibitor that plays a key role in hormone receptor (HR)-positive, human epidermal growth factor receptor 2 (HER-2)-negative breast cancer. However, there have been no reports of acute liver failure in Hepatitis B virus (HBV) carriers caused by HBV reactivation induced by dalpiciclib. We present a 50-year-old female with recurrent breast cancer and a history of asymptomatic HBV carriage. After dalpiciclib initiation, she developed progressive liver impairment, with hepatitis B virus DNA (HBV DNA) escalating from 1.02 × 103 to 2.13 × 10^6^ IU/ml and HBeAg seroconversion from negative to positive, confirming HBV reactivation. Notably, the patient received initial dalpiciclib-based therapy at an external hospital, where antiviral prophylaxis was not initiated despite the availability of baseline HBV serological and viral load data, due to the failure to conduct clinical risk stratification for HBV reactivation based on these results—a critical gap in pre-therapy assessment for HBV-related risk stratification. Entecavir antiviral therapy led to suppressed viral replication and improved liver function during follow-up. This case emphasizes the need for HBV screening and close monitoring of viral markers/liver function in asymptomatic HBV carriers receiving dalpiciclib, as early antiviral intervention prevents severe liver complications. It also highlights the importance of clinical pharmacist-led medication follow-up management for outpatients receiving targeted anticancer therapy to avoid oversight of comorbidities and associated prophylactic treatment.

## Introduction

Breast cancer ranks as the most commonly diagnosed malignancy and the leading cause of cancer-related deaths among women worldwide (1). Hormone receptor (HR)-positive subtypes account for over 75% of all breast cancer cases, with dysregulated cyclin-dependent kinase 4/6 (CDK4/6) signaling being a key driver of tumor proliferation and endocrine resistance (2, 3). As targeted therapies revolutionize the management of advanced or metastatic HR+/human epidermal growth factor receptor 2 (HER2)- breast cancer, CDK4/6 inhibitors (CDK4/6i) have emerged as a cornerstone of treatment, exerting their efficacy by blocking G1-to-S phase cell cycle progression and suppressing tumor growth (4, 5). Dalpiciclib is a novel oral selective CDK4/6i with established activity in heavily pretreated HR+/HER2- advanced breast cancer, both as monotherapy and in combination with aromatase inhibitors or fulvestrant (6–9).

Hepatitis B virus (HBV) is a major global health concern that causes chronic infections (10). Approximately 250 million individuals worldwide are currently living with chronic HBV infection, with a substantial proportion residing in Asia and Africa (11). It is estimated that 15%−40% of infected patients will progress to develop cirrhosis, liver failure, or hepatocellular carcinoma (12); thus, eradicating HBV infection solely through existing therapeutic modalities remains a significant challenge. Previous studies have linked HBV reactivation (HBVr) to chemotherapy in patients with malignant tumors (13–15). Patients experiencing HBVr may be asymptomatic; however, when combined with chemotherapy drugs, HBVr can inflict severe harm (16). Notably, immunosuppressive anticancer agents such as CDK4/6 inhibitors do not typically exhibit direct potent hepatotoxicity but can disrupt the host's immune surveillance of HBV, leading to reactivation of latent infection in carriers and subsequent severe liver injury—a critical distinction from direct drug-induced liver injury (DILI) that has important clinical implications for prophylaxis and management.

To date, there have been no reports of HBVr associated with dalpiciclib treatment; among the adverse reactions (AEs) induced by dalpiciclib, liver failure is also extremely rare. The adverse reactions section in the drug instructions for dalpiciclib does not contain any description related to the liver system. In clinical trials, the most common non-hematological AEs are low-grade liver enzyme abnormalities (5, 9). In a phase III RCT study of dalpiciclib combined with fulvestrant for the treatment of hormone receptor-positive HER2-negative advanced breast cancer, it was mentioned that among 240 patients in the dalpiciclib combined with fulvestrant treatment group, 1 patient (0.4%) experienced hepatic failure and died (9). While existing data suggest that HBV carriers may have an increased risk of hepatotoxicity with CDK4/6 inhibitor use, dalpiciclib-associated HBVr leading to severe liver failure has not been previously reported, representing a critical novel finding of this case report. Herein, we present the first reported case of HBVr-induced liver failure in a breast cancer patient receiving dalpiciclib. Through detailed analysis of clinical manifestations, laboratory findings, and therapeutic interventions, this report aims to raise awareness of this potentially fatal complication, provide practical guidance for clinicians managing HR+/HER2- breast cancer in HBV carriers, and emphasize the role of proactive screening and prophylaxis in mitigating such risks.

## Case Report

A 50-year-old female patient, with a height of 165 cm and a weight of 70 kg, was admitted to the hospital on April 3, 2025. Her medical history included a history of left breast cancer surgery 11 years prior, as well as abdominal distension and skin jaundice for more than 10 days. She was diagnosed with recurrent invasive carcinoma of the left breast after surgery (Stage IV, rTxN3M1) and secondary malignant neoplasm of the supraclavicular lymph nodes. Past Medical History: in 2014, she underwent radical mastectomy for left breast cancer, followed by 6 cycles of postoperative chemotherapy (specific regimen unknown); she had been an asymptomatic HBV carrier for 49 years. In September 2024, the patient presented with disease recurrence; a biopsy of the recurrent lesion revealed adenocarcinoma. Immunohistochemical (IHC) staining results were as follows: estrogen receptor (ER): 3+, progesterone receptor (PR): 3+, HER2: 2+. Subsequent fluorescence *in situ* hybridization (FISH) testing confirmed HER2 negativity (–).

The patient initiated dalpiciclib-based therapy at an external hospital in September 2024. Although her HBV carrier status was known (Hepatitis B surface antigen (HBsAg)-positive with detectable HBV DNA of 1.02 × 103 IU/ml, Table 1), antiviral prophylaxis was not initiated—representing a missed opportunity for prevention. On September 24th, 2024, the patient initiated treatment with dalpiciclib 150 mg orally once daily for 21 consecutive days, followed by a 7-day drug holiday, in combination with letrozole 2.5 mg orally once daily on a continuous basis. Post-treatment, the patient's symptoms improved and lymph nodes regressed. In late March 2025, the patient developed abdominal distension and scleral icterus with skin jaundice without obvious predisposing factors, which gradually worsened accompanied by fatigue. Upon admission, physical examination revealed grade I hepatic encephalopathy manifestations, including mild confusion and slurred speech (17). Laboratory test results on April 3, 2025: HBsAg, Hepatitis B e antigen (HBeAg), and Hepatitis B core antibody (anti-HBc) were positive (detailed results are presented in Table 1). HBV DNA: 2.13 × 10^6^ IU/ml. Alanine Aminotransferase (ALT) 761 U/L, Aspartate Aminotransferase (AST) 744 U/L, Alkaline Phosphatase (ALP) 206 U/L, γ-Glutamyl Transferase (GGT) 122 U/L, Total Bilirubin (TBiL) 336.1 μmol/L, Direct Bilirubin (DBiL) 208.0 μmol/L, international normalized ratio (INR): 3.88, prothrombin time (PT): 42.8 s, D-dimer: 16.59 μg/ml, serum ammonia: 156 μmol/L (reference range: 9–30 μmol/L).

**Table 1:** The result of hepatitis B virus screening for a 50-year-old female before and after treatment with dalpiciclib.

**HBV screening**	**Initial treatment**	**After treatment with dalpiciclib for 5 months**	**After taking entecavir for 3 months**	**Reference range**
HBsAg, IU/ml	1,250	200.31	168.72	< 0.05
Anti-HBs, mIU/ml	2.8	0.66	0.73	< 10.00
HBeAg, S/CO	0.42	13.03	9.45	< 1.00
Anti-HBe, S/CO	3.8	0.6	0.87	>1.00
Anti-HBc, S/CO	8.6	6.46	6.12	< 1.00
HBV DNA, IU/ml	1.02 × 10^3^	2.13 × 10^6^	4.37 × 10^3^	< 100

HBsAg, hepatitis B surface antigen; HBs, Anti-HBs, antibody to hepatitis B surface antigen; HBeAg, hepatitis B e antigen; Anti-HBe, antibody to hepatitis B e antigen; Anti-HBc, antibody to hepatitis B core antigen; HBV, hepatitis B virus.

The patient presented with scleral icterus and cutaneous jaundice involving the face and trunk, along with clinical manifestations of grade I hepatic encephalopathy. Concurrently, laboratory investigations revealed elevated HBV antigen load consistent with HBVr. In addition to the aforementioned manifestations, the patient had recently developed abdominal distension, anorexia, marked hyperammonemia, and abnormalities in liver function tests (LFTs) and coagulation profiles, which collectively met the diagnostic criteria for liver failure. Promptly, the suspected culprit medications (dalpiciclib and letrozole) were discontinued. Antiviral therapy was initiated with entecavir capsules 0.5 mg, once daily [qd], orally [po]. Hepatoprotective and symptomatic supportive treatments were administered for clinical management of severe liver injury, including the following (note: these treatments are used for clinical supportive care at our institution, and evidence from large-scale clinical trials for their efficacy in severe liver failure is limited): Monoammonium glycyrrhizinate cysteine sodium chloride injection 200 mL, qd, intravenous drip [ivgtt] for 4 consecutive days; Magnesium isoglycyrrhizinate injection 200 mg diluted in 0.9% sodium chloride injection 100 ml, qd, ivgtt for 4 days. Furthermore, enzyme-lowering therapy was prescribed with bicyclol tablets 50 mg, three times daily [tid], po, and ursodeoxycholic acid 250 mg, tid, po. Serial LFT monitoring was performed for the patient. Given the abnormal coagulation parameters, which were attributed to coagulopathy secondary to liver dysfunction, close surveillance of coagulation profiles was conducted alongside symptomatic hepatoprotective management.

On April 7, abdominal paracentesis was performed for ascites drainage, leading to a noticeable alleviation of abdominal distension. However, repeat LFTs demonstrated the following results: ALT 324 U/L, AST 287 U/L, ALP 254 U/L, GGT 127 U/L, TBiL 449.2 μmol/L, DBiL 320.2 μmol/L, serum ammonia: 112.7 μmol/L. Although transaminase levels decreased moderately following hepatoprotective therapy, the persistent rise in bilirubin concentrations is a hallmark of “enzyme-bilirubin dissociation”. In the context of acute liver injury, this phenomenon signals a transition from predominantly hepatocellular inflammation to a state of functional hepatic decompensation, characterized by impaired synthetic and excretory capacity. Its clinical significance is underscored by the concurrent presence of hepatic encephalopathy and severe coagulopathy—features that distinguish progressive liver failure from uncomplicated, self-limited liver injury, where such systemic manifestations are absent. Consequently, the patient was transferred to the Intensive Care Unit (ICU) for further aggressive management. On April 8, laboratory tests of coagulation function revealed the following results: INR 5.81; PT 51.8 s, and D-dimer 17.21 μg/ml; additionally, the platelet count dropped to 35 × 10^9^/L. Given the patient's severe thrombocytopenia and coagulation impairment, prompt interventions were initiated to correct the coagulation disorder, including administration of recombinant human thrombopoietin injection, human fibrinogen, and human prothrombin complex concentrate. The patient remained in liver failure. Bilirubin adsorption therapy was promptly initiated. Following treatment, serum ammonia decreased significantly to 62.7 μmol/L. Bilirubin adsorption therapy yielded significant efficacy: TBIL levels decreased from 449.2 to 236.4 μmol/L. Meanwhile, close monitoring of platelet counts and coagulation function was performed throughout the entire treatment course.

On April 9, follow-up LFTs showed: ALT 113 U/L, AST 285 U/L, ALP 131 U/L, GGT 65 U/L, and TBiL 406.5 μmol/L. At this stage, the patient's condition was classified as progressive liver failure with persistently elevated bilirubin. Therefore, plasma exchange therapy was planned in addition to the ongoing hepatoprotective regimen.

**Figure 1:**
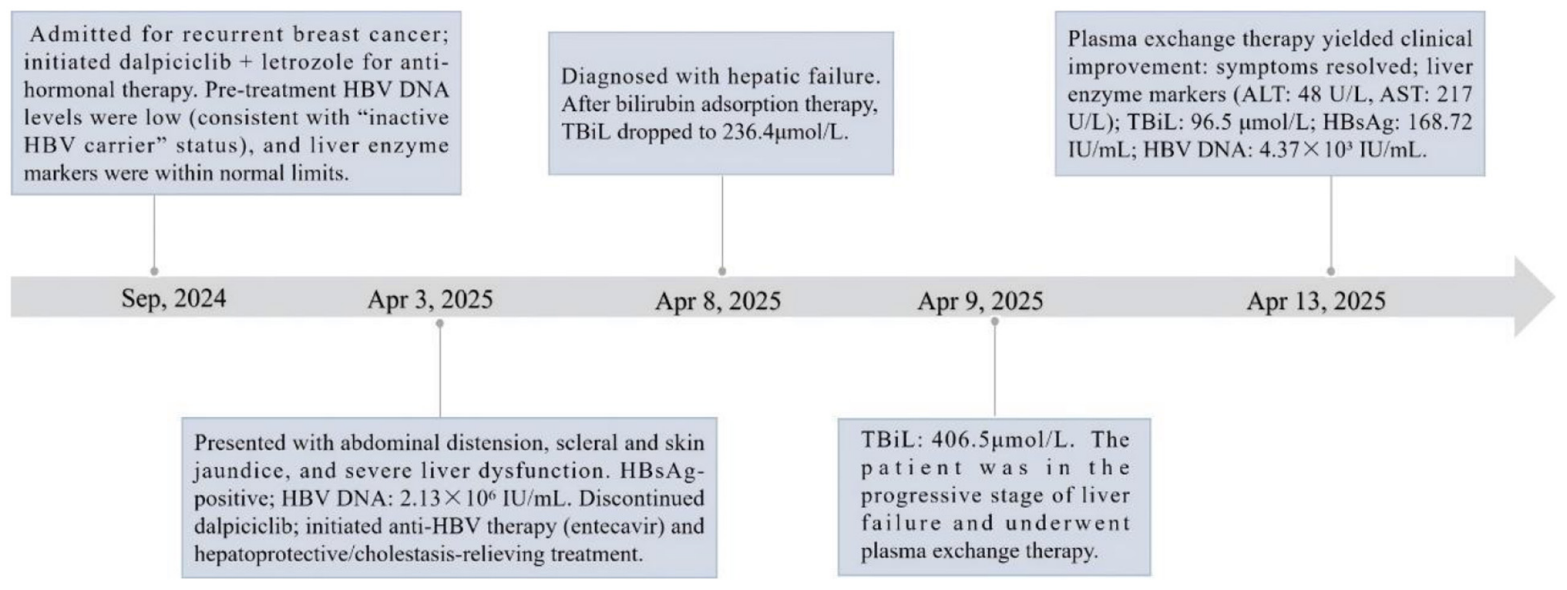
The timeline for the treatment and examination of the patient. HBV, hepatitis B virus; HBsAg, hepatitis B surface antigen; ALT, Aminotransferase; AST, Aspartate Aminotransferase; TBiL, Total Bilirubin.

**Figure 2:**
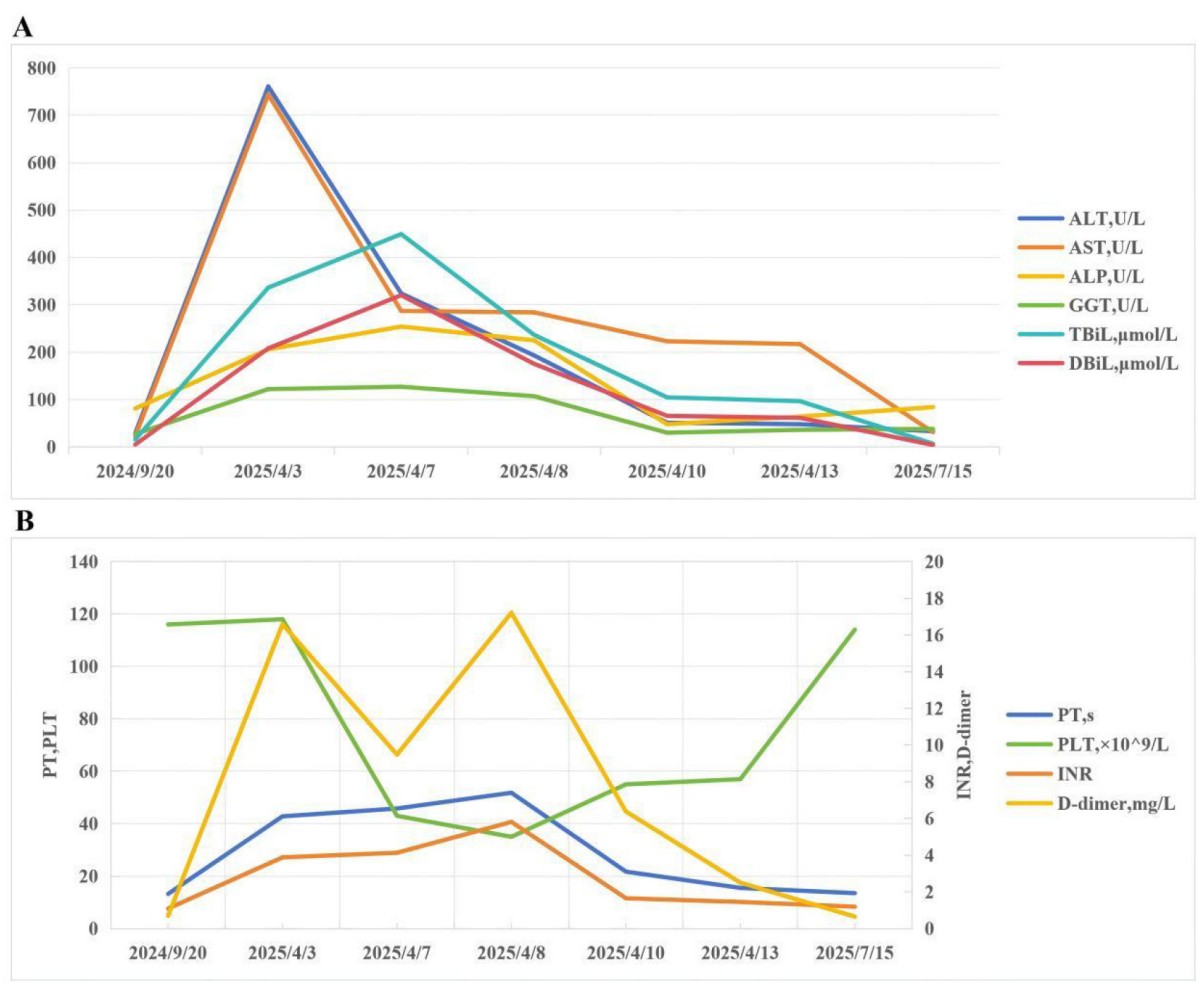
The changes in biochemical and coagulation indicators of the patients: pre-dalpiciclib administration, during this hospitalization, and at the 3-month follow-up. **(A)** The changes in biochemical indicators of the patients. **(B)** The changes in coagulation indicators of the patients. ALT, Aminotransferase; AST, Aspartate Aminotransferase; ALP, Alkaline Phosphatase; GGT, γ-Glutamyl Transferase; TBiL, Total Bilirubin; DBiL, Direct Bilirubin; INR, International Normalized Ratio; PT, Prothrombin Time; PLT, Platelet.

On April 13, repeat LFTs revealed remarkable improvements: ALT 48 U/L, AST 217 U/L, ALP 64 U/L, GGT 36 U/L, and TBiL 96.5 μmol/L. Serum ammonia was normalized at 24 μmol/L, and all hepatic encephalopathy manifestations were completely resolved (no confusion or slurred speech). Since plasma exchange therapy achieved favorable therapeutic outcomes and the patient's clinical condition stabilized, she was successfully discharged from the hospital (Figure 1). The patient was continued to receive antiviral drugs orally: entecavir capsules 0.5 mg, qd, for 6 months. The patient was prescribed oral medications for liver function maintenance: glutathione tablets 400 mg tid po; silymarin capsules 70 mg tid po and ursodeoxycholic acid 500 mg qd po. The three drugs were combined for the maintenance of liver function after discharge. Three months after discharge, the patient was followed up. The patient's liver function and coagulation function had significantly improved, and the HBV DNA level dropped to 4.37 × 10^3^ IU/ml. The changes in biochemical and coagulation indicators of the patients before and after treatment are shown in Figure 2.

## Discussion

HBVr is defined as the reappearance of HBV particles in patients with non-active HBV infection who are suffering from malignant tumors or other diseases (18). For tumor patients with HBV carriage, the presence of underlying liver disease may increase the risk of severe hepatic adverse reactions when exposed to potential hepatotoxic drugs. Cytotoxic agents or immunosuppressive therapies can impair the body's immune surveillance mechanism, leading to elevated serum HBV DNA levels, which in turn promote massive HBV replication and ultimately results in rapid infection of normal hepatocytes (19). Dalpiciclib, a highly selective CDK4/6 inhibitor for HR+/HER2- advanced breast cancer, exhibits minimal direct hepatotoxicity, with its hepatic adverse reactions mainly manifested as mild to moderate asymptomatic transaminase elevation in the early treatment stage; severe liver failure caused by direct drug-induced hepatotoxicity is extremely rare (9). Although dalpiciclib does not directly target the HBV genome, its immunosuppressive effects during tumor treatment can indirectly weaken the immune system's surveillance and control of latent HBV infection, leading to HBV reactivation in carriers. This results in massive viral replication and subsequent hepatocellular injury—this is the primary pathogenic mechanism of liver injury in the present case, rather than direct dalpiciclib-induced DILI (20).

In the present case, the patient received combined therapy with dalpiciclib and letrozole. Dalpiciclib's immunosuppressive effect was the key driver of HBV reactivation, which subsequently caused severe acute liver failure; letrozole has low hepatotoxicity, mainly manifesting as mild elevation of liver enzymes, and no cases of liver failure caused by letrozole have been reported, ruling out letrozole as a contributing factor to the severe liver injury. Dalpiciclib may cause mild increases in ALT and AST; although severe liver failure is relatively rare, cases of liver failure induced by dalpiciclib have been documented in clinical studies, which may be associated with unrecognized HBV reactivation in those cases (9). In addition, cases of severe liver injury have been reported in patients receiving other CDK4/6 inhibitors. For instance, fatal acute liver failure has been documented in breast cancer patients receiving combined therapy with abemaciclib and fulvestrant (21). Moreover, hepatotoxicity has also been reported with ribociclib and palbociclib (13, 22, 23), and existing data suggest that HBV carriers may have an increased risk of hepatotoxicity with CDK4/6 inhibitor use (24)—highlighting a shared class effect of CDK4/6 inhibitors on HBV reactivation risk via immunosuppression, even if dalpiciclib-associated HBVr leading to liver failure is a novel finding in this case. Therefore, the liver failure in this case was primarily driven by HBV reactivation consequent to dalpiciclib-induced immunosuppression. It is worth noting that the patient, as a long-term HBV carrier, may have had a relatively poor baseline liver reserve, which could have rendered her more vulnerable to the significant hepatocellular injury caused by the massive viral reactivation and subsequent immune response. A critical clinical oversight in this case was the failure to initiate antiviral prophylaxis prior to dalpiciclib therapy at the external hospital, which is a core recommendation in the American Association for the Study of Liver Diseases (AASLD) 2018 guidance (25) and The Chinese Guidelines for the Prevention and Treatment of Chronic Hepatitis B (2022 Edition) for patients receiving immunosuppressive or chemotherapeutic anticancer therapy—regardless of whether the agent is explicitly named in guidelines, the immunosuppressive nature of CDK4/6 inhibitors mandates HBV risk stratification and prophylaxis.

To the best of our knowledge, this is the first reported case of HBV reactivation-induced liver failure following dalpiciclib treatment, highlighting that HBVr can be a life-threatening complication of this agent. The exact mechanism underlying the association between dalpiciclib and HBVr remains to be fully elucidated. Current evidence points to immune-mediated liver damage from host immune microenvironment perturbations, rather than direct drug metabolite toxicity. Therefore, caution should be exercised when administering dalpiciclib to patients confirmed as HBV carriers. This case serves as a critical clinical alert: high HBV DNA loads, combined with antineoplastic agent exposure, significantly increase the risk of HBVr in HBV carriers, emphasizing the necessity of proactive preventive strategies in this high-risk population. This case also underscores the importance of clinical pharmacist-led post-discharge medication follow-up management for patients receiving targeted anticancer therapy, as it can identify comorbidities such as HBV carriage and ensure compliance with prophylactic antiviral therapy, avoiding clinical oversights in outpatient settings.

International guidelines regarding pre-chemotherapy HBV screening and prophylactic antiviral therapy for cancer patients exhibit subtle variations. The American Society of Clinical Oncology (ASCO) 2020 guidelines recommend that all patients anticipated to receive systemic anticancer therapy should undergo HBV screening using three markers—HBsAg, anti-HBc total immunoglobulin, and anti-HBs—prior to or at the initiation of therapy, with prophylactic antiviral treatment continued throughout anticancer therapy and for at least 12 months thereafter (26). In contrast, the European Association for the Study of the Liver (EASL) 2025 guidelines categorize cancer patients undergoing chemotherapy as a high-risk group for HBV screening, requiring initial screening with HBsAg and anti-HBc, with positive results necessitating quantitative HBV DNA testing to guide management (27). The AASLD 2018 guidance explicitly recommends initiating antiviral therapy in patients receiving chemotherapy or immunosuppressive medications for cancer, regardless of the specific agent class—this includes CDK4/6 inhibitors such as dalpiciclib, given their immunosuppressive properties that disrupt HBV immune surveillance (25). The Chinese Guidelines for the Prevention and Treatment of Chronic Hepatitis B (2022 Edition) mandate routine screening for HBsAg, anti-HBc, and/or HBV DNA in all patients prior to chemotherapy, targeted therapy, or immunosuppressive therapy. For HBsAg-positive and/or HBV DNA-positive patients, prophylactic antiviral therapy with entecavir (ETV), tenofovir disoproxil fumarate (TDF), or tenofovir alafenamide fumarate (TAF) should be initiated at least 1 week before the start of anticancer therapy (28).

Supporting the importance of proactive screening and prophylaxis, previous studies have demonstrated that serial monitoring of serum HBV DNA levels effectively prevents HBVr in patients with B-cell non-Hodgkin lymphoma and a history of HBV infection (29). Furthermore, a case report described a 45-year-old female with metastatic breast cancer and active HBV infection who achieved favorable outcomes with concurrent ribociclib and tenofovir disoproxil fumarate for HBV eradication (13). Collectively, these findings underscore the imperative for clinicians to meticulously assess HBV serological markers before initiating CDK4/6 inhibitor therapy. For HBsAg-positive and/or HBV DNA-positive patients, a combined approach of curative HBV therapy and oncological treatment for breast cancer is feasible. Prior to initiating therapy in patients requiring a multidisciplinary approach, a comprehensive assessment of the clinical scenario is essential, followed by rigorous long-term follow-up for potential HBVr post-drug initiation. This case adds to the existing literature on CDK4/6 inhibitor-associated hepatotoxicity in HBV carriers by identifying dalpiciclib as a novel agent linked to HBVr-induced severe liver failure, offering practical guidance for pre-therapy risk stratification, prophylaxis, and post-therapy monitoring in this high-risk population.

For the patient in the present case, following dalpiciclib-induced life-threatening liver failure, permanent discontinuation of dalpiciclib is mandatory. For subsequent anticancer therapy, after achieving sustained HBV DNA suppression with antiviral therapy and complete normalization of liver function, a switch to an alternative CDK4/6 inhibitor (e.g., palbociclib, ribociclib, or abemaciclib) may be considered following multidisciplinary team (MDT) discussion (24). Emerging evidence supports the feasibility of switching between CDK4/6 inhibitors after severe hepatotoxicity, with successful transitions documented in multiple reports (30–33). If rechallenge with an alternative CDK4/6 inhibitor is pursued, it should be initiated at a reduced dose with intensive monitoring of liver function and HBV DNA levels (24).

## Conclusion

For patients with HR+/HER2- advanced or metastatic breast cancer treated with dalpiciclib, pre-treatment evaluation of HBsAg and/or HBV DNA levels is mandatory. For those with HBV carriage, concurrent management of HBV infection and breast cancer using curative antiviral therapy and oncological treatment strategies is recommended, with close monitoring of liver function, HBV viral load, and serum ammonia levels throughout the treatment course to detect early signs of HBV reactivation and liver failure. This case confirms that dalpiciclib's severe hepatotoxicity in HBV carriers stems from immunosuppression-driven HBV reactivation, not direct toxicity—underscoring the need for antiviral prophylaxis over passive enzyme monitoring. While a risk-benefit assessment is indispensable for each individual patient, this case report provides valuable insights for clinicians managing patients with significant comorbidities such as chronic HBV infection. It also highlights the vital role of clinical pharmacist-led follow-up in preventing such severe complications.

## Data Availability

The original contributions presented in the study are included in the article/supplementary material, further inquiries can be directed to the corresponding authors.
